# A CRISPR Powered Immobilization-Free, Amplification-Free Carbon-Nanotube Field-Effect Transistor (FET) Sensor for Influenza A Virus (IAV)

**DOI:** 10.3390/molecules30234608

**Published:** 2025-11-30

**Authors:** Wenjun Li, Yue Shi, Dong Li, Yihan Wang, Yansong Sun, Hao Li, Yao Han

**Affiliations:** State Key Laboratory of Pathogen and Biosecurity, Academy of Military Medical Sciences, Beijing 100071, China; liwenjun181@mails.ucas.ac.cn (W.L.); shiyoo22@163.com (Y.S.); zzulidong2007@163.com (D.L.); wangyihan_698@163.com (Y.W.); sunys6443@126.com (Y.S.)

**Keywords:** carbon-nanotube field-effect transistors, CRISPR/Cas13a, IAV, biosensors

## Abstract

The epidemic of infectious diseases, such as influenza A, has imposed a severe health burden on the population. Early detection, diagnosis, reporting, isolation, and treatment are crucial for the prevention, control, and management of infectious diseases. Nucleic acid testing represents a vital approach for the rapid diagnosis of pathogenic microorganism types. However, current nucleic acid detection methods face notable bottlenecks: traditional CRISPR fluorescence assays require time-consuming pre-amplification of target nucleic acids, while existing carbon-nanotube field-effect transistor (FET)-based platforms, though amplification-free, often necessitate complex chip surface modification and probe immobilization, and suffer from non-reusable chips, all limiting their utility in point-of-care testing (POCT) and large-scale screening. This study reports a CRISPR-based amplification-free RNA detection platform (CRISPR-FET) for the rapid identification of influenza A virus. The CRISPR-FET platform described herein enables the detection of viral RNA without amplification within 20 min, with a limit of detection as low as 1 copy/μL. Secondly, a reporter RNA conjugated with gold particles is used to achieve signal amplification in FET detection; meanwhile, the method eliminates probe immobilization, thereby omitting this step and simplifying chip modification to reduce complex work-flows and pre-treatment costs. The chip’s reusability further enhances cost-effectiveness. Additionally, streptavidin-modified magnetic bead adsorption minimizes background errors from excessive reporter RNA and non-target nucleic acids. Finally, validation with 24 clinical samples confirmed the platform’s efficacy. By integrating rapidity, simplicity, and high sensitivity, alongside cost advantages from reusable chips, this CRISPR-FET platform meets the critical need for early influenza A diagnosis and holds promise for advancing POCT and large-scale epidemiological screening.

## 1. Introduction

Influenza A virus (IAV) is a single-stranded negative-sense RNA virus that infects a broad spectrum of avian and mammalian species, including humans. It is one of the principal pathogens causing human influenza pandemics [1]. IAV can cause seasonal influenza and even global pandemics in humans, posing a severe threat to public health. Seasonal influenza viruses cause approximately 3 to 5 million severe infections and 290,000 to 650,000 deaths worldwide each year [2,3]. For infectious disease prevention and control, the key principles are early prevention, detection, diagnosis, treatment, and isolation. Due to overlapping symptoms with other respiratory pathogens, clinical diagnosis of IAV is often inconclusive, making pathogen detection crucial for clinical decision-making [4]. Current major methods for IAV detection include nucleic acid testing, immunology-based assays, and viral culture [5,6,7]. Viral culture remains the traditional gold standard for pathogen detection, renowned for its broad applicability, low cost, and high accuracy. However, this method is excessively time-consuming, requiring several days to obtain results [8]. Immunological assays detect pathogens flexibly via antigen–antibody interactions, significantly reducing testing time. Rapid antigen tests yield results within minutes, but their low sensitivity often restricts confirmation to the stage when symptoms emerge and viral shedding begins, limiting effectiveness in epidemic prevention and control [6,9].

Nucleic acid testing is an experimental method for identifying specific pathogens or genetic information by detecting DNA or RNA in biological samples [10]. This approach features high sensitivity and specificity, with a detection limit as low as 1 copy per microliter. It requires only a few hours for results and enables early pathogen detection during the infection stage [11]. Current nucleic acid detection methods are primarily categorized into polymerase chain reaction (PCR)-based techniques (such as quantitative real-time PCR (qPCR) and digital PCR) and isothermal nucleic acid amplification methods [12]. As the gold standard in clinical nucleic acid testing, qPCR is widely used but requires approximately 1 h to perform, and its reliance on temperature-control equipment limits its application in point-of-care testing (POCT). Digital PCR offers high accuracy and reproducibility in pathogen detection, enabling absolute quantification without calibration curves, but is limited by high costs, complex operation, and limited sensitivity and dynamic range [13,14]. Isothermal amplification techniques amplify target nucleic acid concentration through enzymatic reactions at a constant temperature, using colorimetric or fluorescent indicators for result visualization, thus eliminating the need for complex temperature-control devices [15,16]. Biosensors based on isothermal amplification have also been developed for the detection of specific pathogens [17]. However, both PCR and isothermal amplification methods require nucleic acid amplification, which involves specific primer design, complex enzymatic reactions, and multiple operational steps, and may lead to false-positive results due to contamination [15].

The Clustered Regularly Interspaced Short Palindromic Repeats (CRISPR) system, as a third-generation gene-editing tool, has demonstrated excellent performance in nucleic acid detection [18]. It has been widely applied to detect pathogens of various infectious diseases, including influenza, COVID-19, and monkeypox, gaining increasing recognition in the field of point-of-care nucleic acid testing [19,20]. The CRISPR-Cas13a system contains two conserved Higher Eukaryotes and Prokaryotes Nucleotide-binding (HEPN) domains, endowing it with ribonuclease activity. These domains enable Cas13a to catalyze the formation of crRNA and RNA-guided degradation of single-stranded RNA (ssRNA) through two independent catalytic sites. Cas13a binds to precursor-crRNA (pre-crRNA) to form a crRNA-Cas13a complex [21]. The guide region of crRNA recognizes target RNA via a complementary sequence, activating Cas13a. This activation triggers specific cleavage of the target RNA and non-specific trans-cleavage of non-target RNAs. The trans-cleavage activity of the CRISPR-Cas13a system can effectively amplify biological signals by recognizing target sequences using crRNA and trans cleaving reporter RNA in the reaction system [22].

Carbon-nanotube Field-effect Transistors (FETs) are solid-state semiconductor devices composed of a source, drain, and gate, utilizing the electric field effect to control the current of the transistor [23]. At present, there are few graphene field-effect transistor biosensors combined with CRISPR-Cas13a, all of which have been applied to detect SARS-CoV-2 RNA or its variants [24]. Li et al. also reported a non-amplifying nucleic acid detection biosensor using the trans cleavage mechanism of Cas13a and an ultrasensitive graphene field-effect transistor (GFET), which can detect SARS-CoV-2 RNA as low as 1 aM within 30 min [25]. However, this method recognizes target molecule signals through the degree of Dirac point shift, which can also cause signal interference over time [25]. Sun et al. have found that hydrophobic treatment of graphene FET substrates can effectively address this instability [26]. The graphene field-effect transistor biosensor, combined with CRISPR-Cas13a reported by Sun et al., provides a high-intensity vector signal for detecting SARS-CoV-2. SARS-CoV-2 RNA generates a “large subtraction” signal with right shift characteristics, while any non-target will generate a left shift characteristic signal. This method can detect SARS-CoV-2 RNA as low as 0.25 aM [26]. But this method requires 2 h for detection, and the sensitivity has decreased slightly [26]. FET biosensors have high sensitivity for detecting biomolecules, but in actual samples with high ion concentrations, ion screening effects, and various interferences reduce the performance of the sensor. The combination of CRISPR detection technology for biological signal amplification and the highly sensitive recognition ability of FETs in biosensors is expected to facilitate the development of a new type of highly sensitive and specific on-site detection technology [27]. The study of stability, repeatability, and reproducibility has always been an essential component of the development of FET biosensors [28]. The current FET biosensor detection platform based on CRISPR-Cas13a detects by immobilizing the reporter RNA on the surface of the device [29,30,31]. The method of fixing the reporter RNA is operationally complex, and the direct fixation method affects the reuse of the sensor chip. The method of treating the surface of the device and then fixing the reporter RNA achieves the reuse of the device while greatly increasing the detection time and reducing sensitivity. The current challenge is to develop a reusable, fast, highly sensitive, and specific field-effect transistor biosensor. Currently, DNA probes represent the most commonly used biorecognition elements in FET-based nucleic acid detection. These probes, functioning as molecules that specifically recognize target sequences, are integrated with FETs to form biosensors. Typically, in DNA-FET systems, DNA probes complementary to the target sequence are immobilized on the surface of the conductive channel. When hybridization occurs between the DNA probe and the target sequence, the introduction of additional negative charges induces a change in surface potential, which in turn alters the threshold voltage and source-drain current of the field-effect transistor [32].

This study developed an amplification-free carbon-nanotube field-effect transistor biosensor based on CRISPR-Cas13a and carbon-nanotube field-effect transistor (CRISPR-FET), which enables rapid, highly sensitive, and specific on-site detection technology through non-fixed reporter RNA. CRISPR-FET recognizes target viral RNA via sequence-specific recognition by crRNA, thereby activating the cleavage activity of Cas13a. This activated Cas13a cleaves the reporter molecule, biotin-ssRNA-AuNPs, which is a 20-nucleotide (nt) ssRNA (poly U) modified with biotin at the 5’end and AuNPs at the 3’end. In positive samples, the cleavage of the reporter molecule releases Au particles into the FET detection system. In contrast, in negative samples, the reporter molecule remains intact without being cleaved by the protein; these intact molecules are adsorbed by streptavidin-labeled magnetic beads, leaving no Au-labeled reporter molecules in the FET detection system. The field-effect transistor (FET) sensor then generates electrical signals based on these differences. Leveraging the inherent electrical signal transduction capability of FET, the recognition signal is amplified, enabling amplification-free and highly sensitive detection of viral RNA within 20 min. CRISPR-FET can achieve target viral RNA detection as low as 1 copy per microliter, approaching the sensitivity of RT-PCR. This platform provides a new solution for the integration of carbon nanofield-effect transistor biosensors and CRISPR-Cas13a technology, and provides a foundation for the development of carbon nanofield-effect transistor biosensors.

## 2. Results and Discussion

### 2.1. Design of FET Biosensor

**Figure 1:**
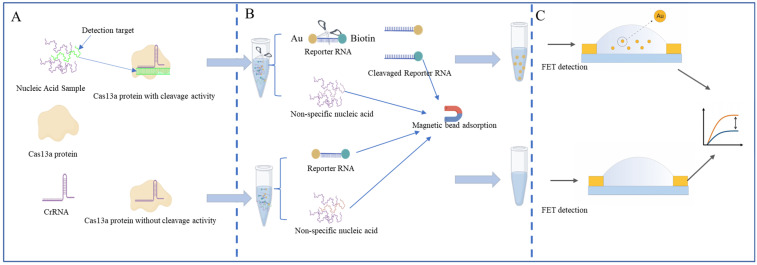
Schematic diagram of CRISPR-FET (**A**) Activation of Cas13a trans-cleavage activity (**B**) Streptavidin magnetic beads adsorbing reporter molecules and nucleic acids (**C**) FET chip detecting gold particle signals.

The principle of the FET biosensor developed is shown in Figure 1, which recognizes target RNA, such as IAV RNA, through CRISPR RNA (crRNA). After recognizing the target RNA, Cas protein activates trans-cleavage activity, resulting in the cleavage of reporter RNA (reRNA), which is a 20-nucleotide (nt) ssRNA (poly U) modified with biotin at the 5’end and AuNPs at the 3’end (referred to as biotin ssRNA AuNPs). After the trans-cleavage activity is completed, SA-coated magnetic beads are used to bind to rRNA through biotin moieties, and any undigested reporter RNA and biotin oligonucleotides in the reaction solution are removed by magnetic separation, leaving AuNPs oligonucleotides in the solution. Subsequently, using carbon-based biological workstations and carbon nanotube chips to detect electrical signals from AuNPs in the mixture, ultrasensitive and specific detection of target RNA was achieved. crRNA recognizes the target nucleic acid and activates the trans-cleavage activity of Cas13a, leading to RNA cleavage. RNA is cleaved into AuNPs, oligonucleotides, and biotin oligonucleotides. The remaining uncleaved RNA and biotin oligonucleotides were removed by coated magnetic beads, leaving AuNPs oligonucleotides in the solution. At this stage, the FET sensor records the electrical signal of AuNPs present in the solution. On the contrary, in the absence of target IAV RNA, FET sensors record the electrical signal of the absence of AuNPs in the solution. This new electrochemical CRISPR biosensor can be used as a low-cost, easily to manufacture, and ultra-sensitive biosensor for detecting IAV and other pathogens.

### 2.2. Validation of CRISPR-FET Biosensors

**Figure 2:**
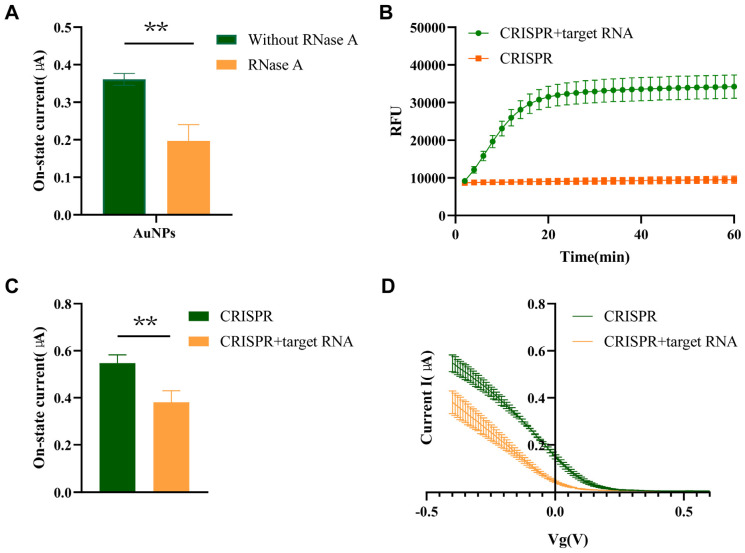
Validation of reRNA effectiveness. (**A**) Changes in on-state current caused by free gold particles after reporter RNA is treated with RNase A; (**B**) Real-time fluorescence curves of groups with and without target nucleic acid within 60 min of the fluorescence-based CRISPR-Cas13a assay; (**C**) Transfer curves detected by FET sensors for groups with and without target nucleic acid; (**D**) On-state current values detected by FET sensors for groups with and without target nucleic acid 1, **: *p* < 0.01. NC: Negative control (detection template: nuclease-free water); Optimization of reaction conditions (*n* = 3).

To validate the feasibility of this CRISPR-FET biosensor, Streptavidin (SA)-coated magnetic beads were used to enrich the number of trans-cleavage-related FET sensor active signal molecules (AuNPs). The separation efficiency of SA coated magnetic beads for reporter RNA was evaluated by measuring the electrical signals of AuNPs and reporter RNA in the solution, as well as the reporter RNA solution treated with SA coated magnetic beads (both prepared 0.1 × PBS solution), and comparing them with the signals obtained from negative control (NC, solution without RNase A added) (Figure 2A). A statistically significant difference in electrical signals between AuNPs and NC (*p* < 0.05) indicates that the presence or absence of AuNPs in 0.1 × PBS solution can produce significant differences in FET electrical signals. To further validate the feasibility of using FET biosensors for nucleic acid detection, we chose the M region gene of IAV as the target RNA. The trans-cleavage activity of Cas13a is activated in the presence of target RNA. By real-time fluorescence monitoring (Figure 2B) and FET biosensor testing (Figure 2C,D), a significant fluorescence signal or current value can be read to monitor the process of reporter RNA cleavage. In the absence of Cas13a, crRNA, or 10,000 copies per microliter of target RNA, there was almost no change in fluorescence and electrochemical signals (Figure 2B–D). After CRISPR/Cas13a trans cleavage activity, the open-state current value of the Cas13a + crRNA + target RNA group was significantly reduced compared to the open-state current value of the Cas13a + crRNA group, indicating that the detection platform has good feasibility.

### 2.3. Screening of High-Efficiency crRNA

**Figure 3:**
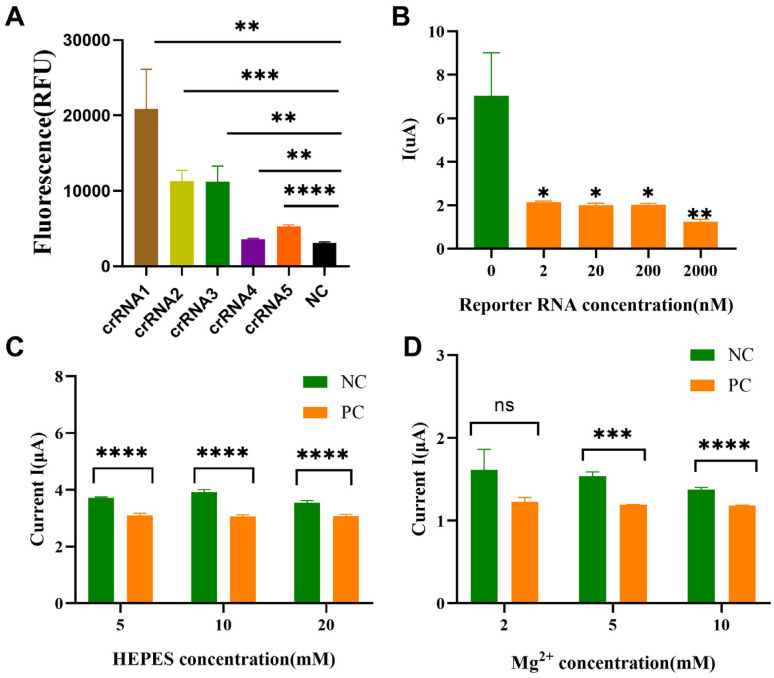
(**A**) Fluorescence values of each group at the 60 min time point in the fluorescence-based CRISPR-Cas13a assay. Student’s *t*-test or Welch’s *t*-test was performed between the fluorescence values of groups crRNA1–5 and the NC (**B**) On-state current values of samples detected by CRISPR-FET under conditions of different magnesium ion concentrations in the system. (**C**) On-state current values of samples detected by CRISPR-FET under conditions of different HEPES concentrations in the system. (**D**) On-state current values of samples detected by CRISPR-FET with different concentrations of reporter RNA molecules. ****: *p <* 0.0001, ***: *p <* 0.001, **: *p <* 0.01, *: *p <* 0.05, ns: not significant. NC: Negative control (detection template: nuclease-free water); Optimization of reaction conditions (*n* = 3).

As depicted in Figure 3A, the relative fluorescence unit (RFU) values of the crRNA1–5 groups at 60 min were 20,892 ± 5235, 11,323 ± 1393, 11,202 ± 2088, 3590 ± 94, and 5292 ± 192, respectively. Each group showed statistically significant differences compared to the NC group (3112 ± 130, *p* < 0.05). Among them, the crRNA1 group demonstrated the highest fluorescence intensity at 60 min, indicating that the Cas13a protein exhibited the highest in vitro reporter RNA cleavage efficiency in the presence of crRNA1. Therefore, crRNA1 was selected for subsequent experiments. Due to the use of non-fixed reporter RNA in this study, the components in the CRISPR system also had an impact on the results. Referring to previous studies on CRISPR-Cas13a and FET sensors, divalent Mg^2+^ ions can affect the cleavage activity of Cas13a. Taking the 10 mM Mg^2+^ concentration used in the CRISPR fluorescence assay as a reference, the Mg^2+^ concentration was optimized to explore whether reducing the Mg^2+^ concentration in the FET assay could improve the FET detection performance. A concentration gradient of 0–10 mM Mg^2+^ was analyzed to verify this effect and it was found that 10 mM Mg^2+^ exhibited the optimal detection performance. At the same time, concentration gradient validation was also conducted on other components in the CRISPR system. Considering cost-effectiveness and practicality, 20 mM HEPES and 2 nM reporter RNA were selected for CRISPR-FET nucleic acid detection.

### 2.4. Sensitivity Detection and Specificity Detection Results

**Figure 4:**
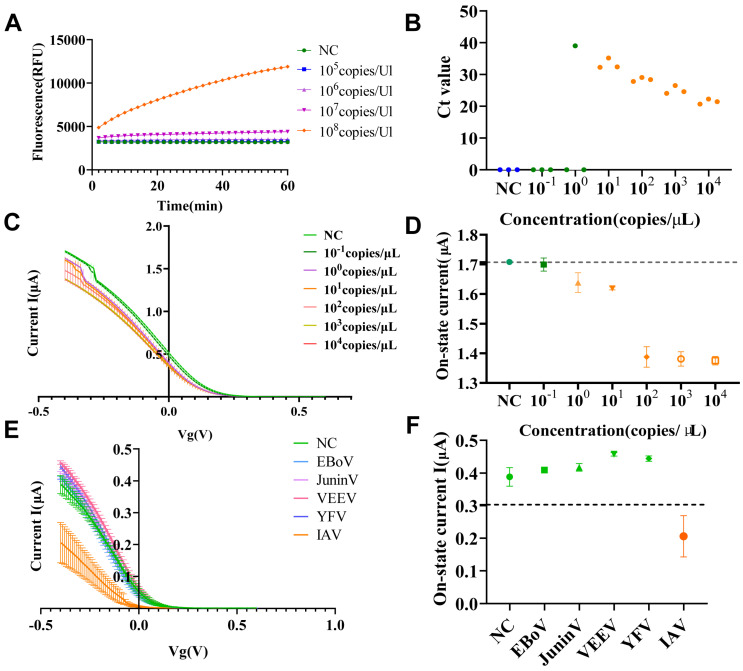
(**A**) Real-time fluorescence detection of non-amplified nucleic acids within 60 min. (**B**) Ct values of RT-qPCR for nucleic acid samples with different concentrations. (**C**) Transfer curves detected by FET sensors for nucleic acid samples with different concentrations. (**D**) On-state current values detected by FET sensors for nucleic acid samples with different concentrations NC: Negative control (detection template: 0.01 × PBS; *n* = 3). Time optimization of reaction conditions. Student’s *t*-test or Welch’s *t*-test was performed between the fluorescence values of the 10^2^ copies/μL, 10^1^ copies/μL, 10^0^ copies/μL groups and the NC, respectively. (**E**) Transfer curves of IAV, EBoV, JuninV, VEEV, and YFV, and NC groups detected by the CRISPR-FET biosensor. (**F**) On-state current values derived from the transfer curves of each group via CRISPR-FET biosensor detection. NC represents the negative control (detection template: nuclease-free water); the black dashed line indicates the positive detection threshold, with *n* = 3. The positive threshold (Cut-off Value) is defined as the average on-state current of the three NC groups minus three times the standard deviation.

To validate the sensitivity of the CRISPR-FET biosensor for influenza A virus detection, this study conducted FET-based assays using influenza A virus nucleic acids at concentrations of 10^4^ copies/μL, 10^3^ copies/μL, 10^2^ copies/μL, 10^1^ copies/μL, 10^0^ copies/μL, and 10^−1^ copies/μL as templates, with enzyme-free water employed as the negative control template. In the present experiment, the positive threshold was determined to be 1.7 μA, calculated by subtracting three times the standard deviation (0.001 μA) from the mean on-state current (1.71 μA) derived from the transfer curves of the negative control group. A detection result was defined as positive if the on-state current measured by the FET sensor was less than 1.7 μA, and negative if the current was greater than or equal to 1.7 μA. As illustrated in Figure 4, when the target nucleic acid concentration was ≥10^0^ copies/μL, the on-state currents detected by the FET sensor were all below 1.7 μA, corresponding to positive results. Conversely, when the target RNA concentration was 10^−1^ copies/μL, the on-state current measured by the FET sensor exceeded 1.7 μA, resulting in a negative outcome. These results demonstrate that the limit of detection (LOD) of the proposed method was 10^0^ copies/μL.

To verify the specificity of the CRISPR-FET biosensor, this study detected nucleic acids of five different pathogens (IAV, Ebola virus (EBoV), Junín virus (JuninV), Venezuelan equine encephalitis virus (VEEV), Yellow Fever virus (YFV)) at a concentration of 10^4^ copies/μL, using nuclease-free water as a negative control. As shown in Figure 4F, the positive threshold was calculated as 0.35 μA, which was calculated by subtracting three times the standard deviation (0.02 μA) from the mean on-state current (0.43 μA) of the NC. The on-state current of the positive control group (PC) was less than 0.35 μA, which was determined to be positive, while the on-state currents of the other pathogen groups and the NC were greater than 0.35 μA, which were determined to be negative. The results showed that the CRISPR-FET biosensor only gave positive results when detecting IAV nucleic acid, while all results were negative when detecting non-IAV nucleic acids, indicating that this method exhibited no cross-reactivity with the other four pathogens under the experimental conditions and further demonstrating its good specificity.

### 2.5. Clinical Sample Detection Results

**Figure 5:**
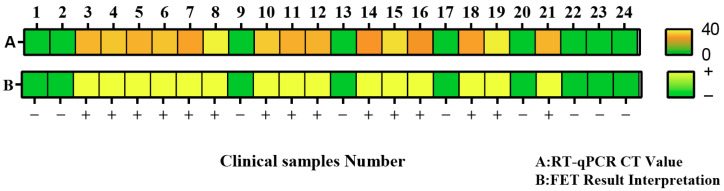
The heatmap shows the detection results of 24 clinical samples. Row A represents the qRT-PCR results of the samples, while Row B represents the results interpreted by the FET detection method.

To further evaluate the performance of this method in detecting clinical samples, 24 human throat swab clinical samples from the Clinical Laboratory of Shanxi Provincial Hospital of Traditional Chinese Medicine were used in this study. All throat swab samples were subjected to nucleic acid extraction using the Magnetic Bead-Based Viral Nucleic Acid Extraction Kit (Catalog No.: AU17011, Beijing Biotech Co., Ltd., Beijing, China) immediately after collection, followed by RT-qPCR and CRISPR-FET detection. The RT-qPCR detection results of clinical samples in this study are shown in Table 1, and the detection results of 24 clinical samples by the CRISPR-FET method are shown in Figure 5. Three groups of negative controls (with nuclease-free water as the detection template) were used for each detection to determine the positive threshold. Among them, the on-state current values of samples 3–7, 10–12, 14, 16, 18, and 21 were lower than the positive threshold, determined as positive, which were consistent with the RT-qPCR results. The CT values of samples 8, 15, and 19 in RT-qPCR results were 38.31, 35.71, and 37.78, respectively, all greater than 35, indicating weak positive samples. Their on-state current values were lower than the positive threshold and determined as positive, suggesting that the CRISPR-FET biosensor can detect weak positive samples. The on-state current values of samples 1–2, 9, 13, 17, 20, 22–24 were greater than the positive threshold, determined as negative. In RT-qPCR results, these samples had CT values greater than 40, indicating negative. The detection results of the CRISPR-FET biosensor for 24 clinical samples were consistent with those of RT-qPCR. Throat swab samples from patients with respiratory tract infections were collected from Shanxi Provincial Hospital of Traditional Chinese Medicine. This study was approved by the Medical Ethics Committee of Shanxi Academy of Traditional Chinese Medicine (Approval No.: 2025KY-08011). All participants provided written informed consent in accordance with the Declaration of Helsinki.

## 3. Materials and Methods

### 3.1. Materials

The RNA reporters in this study (FAM-UUUUUUUUUUUUUUUUUUUU-BHQ-1, Bio-UUUUUUUUUUUUUUUUUUUU-SH) were synthesized by Sangon Biotech Co., Ltd. (Shanghai, China). Gold nanoparticles (AuNPs) were conjugated with thiol groups via the freeze–thaw method to form AuNP-linked reporter RNAs. In contrast, other oligonucleotides, including primer sets and crRNA, were synthesized by Beijing Tianyi Huiyuan Biotechnology Co., Ltd. (Beijing, China) (Table S1). The carbon nanotube-based chip was purchased from Hunan Yuanxin Sensing Technology Co., Ltd. (Xiangtan China). AuNPs were purchased from Nanjing Dongna Biotechnology Co., Ltd. Dynabeams M-280 streptavidin was purchased from Invitrogen (Carlsbad, CA, USA). LwaCas13a protein (Cas13a protein expressed by Leptotrichia wadei) was purchased from GenScript (Nanjing, China). Magnesium chloride (MgCl_2_) was purchased from Shanghai Guoyao Chemical Reagent Co., Ltd. (Shanghai, China). HEPES buffer was purchased from GIBCO (Invitrogen).

### 3.2. CRISPR/Cas13a Fluorescence Assay

Fluorescence detection in this section was performed to screen for highly efficient crRNA for subsequent detection. For the preparation of the CRISPR fluorescence detection system, it is necessary to ensure that the RNase inhibitor is added before to the crRNA, and the reporter molecule is added last during the system preparation process. After the system is prepared, the working concentrations of each component are as follows: HEPES 20 mM, ribonucleoside triphosphate (rNTP) 2 mM, LwCas13a 40 nM, RNase inhibitor 4 IU/µL, crRNA 120 nM, MgCl_2_ 10 mM, T7 RNA polymerase 2 IU/µL, fluorescent-quenched RNA reporter 200 nM, nucleic acid amplification product 5 µL, total reaction volume 25 µL. After the system preparation is completed, mix the solution by pipetting up and down. For real-time fluorescence monitoring, the reaction was carried out in the LightCycler ^®^ Instrument (Roche, Switzerland) at 37 °C for 60 min. Measure the fluorescence of the trans-cleavage reaction every 2 min, with an excitation wavelength of 495 nm and an emission wavelength of 520 nm. Efficient crRNAs were screened by evaluating the fluorescence intensity at 60 min.

### 3.3. Immunomagnetic Bead Separation

For the carbon-nanotube field-effect transistor biosensor experiment of trans-cleavage reporter RNA, the previously described fluorescence-based CRISPR assay was used for the cutting experiment with slight modifications. The working concentration of the components used in the FET CRISPR activation experiment is HEPES 25 mM, Cas13a 75 nM, crRNA 50 nM, biotin-ssRNA-AuNPs 1 µM, MgCl_2_ 25 mM, nucleic acid 5 µL, nuclease-free water, final volume 50 µL. The mixture was incubated at 37 °C for 15 min.

After thorough mixing, pipette 50 µL of SA-coated magnetic bead suspension into a tube and place it on a magnetic stand for 1 min [33]; Discard the supernatant. Resuspend the SA-coated magnetic beads in 40 µL ddH_2_O, place them on the magnetic bracket again for 1 min, and then discard the supernatant. Add 25 µL of CRISPR-FET reaction mixture to the SA-coated magnetic beads for mixing. Finally, place the microcentrifuge tube on a magnetic stand for 1 min, and then collect the supernatant for FET sensor detection.

### 3.4. CRISPR-FET Experiment

FET testing was performed using a carbon-based biological workstation (Hunan Yuanxin Sensing Technology Co., Ltd.). In the transfer curve test, 8 µL of the supernatant was pipetted into the chip sample slot and scanned with a sensor. The source-drain voltage is 0.1 V, and the scanning gate voltage range was −0.4 V to 0.6 V. The supernatant was dropped into the sample tank for 1 min, and tested using an FET sensor. Finally, the transfer curve mode of the FET sensor was selected, with the GateV-begin value set to −0.4 V and the GateV-end value configured to 0.6 V. The current corresponding to a gate voltage of −0.4 V was recorded as the open-state current. After obtaining the open-state current values, data analysis was performed. Used the cut-off method to evaluate the test results. The cut-off current value was determined as positive and negative results by subtracting three times the standard deviation (SD) of the NC group from the average current value.

### 3.5. RT-qPCR Experiment

To compare the detection limits of different methods, the RT-qPCR kit (Nanjing Nuoweizan Biotechnology Co., Ltd., Nanjing, China) was used for reverse transcription quantitative real-time PCR (RT-qPCR). The RT-qPCR reaction system and PCR conditions can be found in the kit instructions. A cycle threshold (Ct) value ≤ 40 is defined as a positive result, while a Ct value > 40 is defined as a negative result.

### 3.6. Sensor Characterization

In this study, the FET chips were purchased from Hunan Yuanxin Sensing Technology Co., Ltd., with the model designated as MC-PB05. The core structural parameters of the FET chips are as follows: the channel length and width are 20 μm × 40 μm, respectively; the areal density of carbon nanotubes (CNTs) in the channel region is approximately 35 tubes/μm^2^. The gate adopts a floating gate structure, and the chip surface is passivated with SU8 material to enhance stability and anti-interference capability. Before the test, the workstation was calibrated and stabilized using a standard resistor. Sample detection was conducted only after the workstation achieved a stable state, so as to ensure the reliability of the test data.

### 3.7. Statistical Analysis

Graphs were plotted using GraphPad Prism 9.0 software. Before comparing the two groups of data, normality analysis was performed via the Shapiro–Wilk test, and homogeneity of variance analysis was conducted using the Levene test. If the data satisfied the normal distribution and homogeneity of variance, Student’s *t*-test (independent samples) was used to analyze intergroup differences; if the normal distribution or homogeneity of variance was not satisfied, the Mann–Whitney U test (nonparametric test) was adopted. This study only involved comparisons between two groups, and no multiple comparisons were performed, thus no multiple test correction was required.

## 4. Discussion

In this study, an amplification-free nucleic acid detection platform based on CRISPR and FET was established for the detection of IAV. We first optimized the components of the reaction system and their ratios, and then evaluated the sensitivity and specificity of this method. The sensitivity of this platform reaches 1 copy/μL. Specificity evaluation was conducted using four other pathogens, and the platform can specifically recognize IAV nucleic acids. It features rapid detection speed and extremely high sensitivity, which benefits from the advantages of FET sensors, such as their ability to convert changes in biological signals into electrical signals, along with rapid response and high sensitivity [34]. They can transduce weak biological signals into measurable electrical signals, thus holding significant potential in the construction of ultra-sensitive biosensors [35]. Its high specificity stems from the CRISPR system with single-base resolution. Currently, FET biosensor detection platforms based on the CRISPR system typically achieve detection by immobilizing probes on the surface of devices [30,35,36]. This probe immobilization method is cumbersome, and the direct immobilization approach affects the reusability and shelf life of sensor chips. Additionally, when conducting multi-target detection, it is necessary to address the issue of spatial isolation for aptamers specific to different targets [37,38]. In this detection platform, CRISPR-Cas13a can accurately recognize target nucleic acids in a homogeneous solution and cleave the reporter RNA, rather than performing cleavage at the interface between the electrode chip and the detection solution. This eliminates the need for immobilizing probes on the chip surface, making the biosensor easy to manufacture and operate while saving time overall. Impurities or non-target molecules in samples may interfere with signals, leading to false positives or reduced sensitivity [39,40]. Therefore, reducing the complexity of the detection system can improve the accuracy of detection. Immunocapture magnetic beads can reduce signal variations caused by background nucleic acids, and at the same time, enable the free gold particles generated by the cleaved reporter RNA in the positive group to be fully distributed in the detection system. In addition, due to the programmability of crRNA, CRISPR-FET biosensors can be developed into a universal biosensing platform applicable to any target RNA by simply altering the sequence of the crRNA guide region, representing a promising amplification-free nucleic acid detection strategy for clinical on-site detection. Biosensors that integrate the high specificity of the CRISPR system with the high sensitivity of FET-based signal detection are expected to evolve into a new type of on-site detection technology characterized by high sensitivity, high specificity, and miniaturization. A potential limitation of this study is that the cross-reactivity panel did not include common respiratory viruses that co-circulate with influenza A virus (IAV) in clinical settings, such as influenza B virus (IBV), respiratory syncytial virus (RSV), and severe acute respiratory syndrome coronavirus 2 (SARS-CoV-2). This may restrict the direct extrapolation of the assay’s specificity to complex clinical scenarios involving multiple potential pathogens. However, in the clinical sample testing of this study, the assay demonstrated good specificity, as evidenced by the absence of positive signals in nucleic acid samples from patients clinically diagnosed with COVID-19. In future validation experiments, we will expand the range of viruses included in the specificity tests to further confirm the specificity of this assay.

Although this study has made efforts in the direction of label-free chips, it also has several limitations. It cannot directly detect clinical samples. Complex clinical samples (such as blood or saliva) may affect signal stability due to ionic strength or protein adsorption [40,41]. Most existing FET detections are based on extracted nucleic acid samples; exploring more efficient nucleic acid release methods will lay a foundation for the subsequent clinical application of FET detection. Secondly, there is no linear relationship between the change in on-state current and the amount of nucleic acid in the sample. Consequently, the detection method involved in this study can only achieve qualitative analysis rather than quantitative analysis. In subsequent research, we will optimize the device structure, improve the detection system, collect a large amount of data, explore the relationship between the current signal and the quantity of the target analyte, and investigate the feasibility of quantifying the target virus in the sample. In addition, the batch of chips and their manufacturing processes can affect their current values and consistency. Therefore, strict negative controls need to be set up for each test, and the standardized production of devices is also a key direction for the future development of FET detection. Finally, although FET devices are easy to miniaturize and suitable for the development of portable diagnostic equipment, they do have issues such as poor stability and oversensitivity in signal acquisition, making them unsuitable for use in unstable environments. Future research will explore more stable applicability. This will lay a foundation for better adaptation to point-of-care testing scenarios in subsequent studies.

## Figures and Tables

**Table 1:** The quantitative fluorescence PCR detection results of influenza A virus (IAV) and clinical interpretation.

Sample ID	Sample Type	RT-qPCR Ct Value	Interpretation Result	Presence of Other Pathogens
1	Throat Swab	-	(−)	
2	Throat Swab	-	(−)	
3	Throat Swab	30.93	Flu A (+)	
4	Throat Swab	32.78	Flu A (+)	
5	Throat Swab	30.4	Flu A (+)	
6	Throat Swab	32.62	Flu A (+)	
7	Throat Swab	28.7	Flu A (+)	SARS-CoV-2 (+)
8	Throat Swab	38.31	Flu A (+)	
9	Throat Swab	-	(−)	SARS-CoV-2 (+)
10	Throat Swab	33.34	Flu A (+)	
11	Throat Swab	30.18	Flu A (+)	
12	Throat Swab	30.54	Flu A (+)	
13	Throat Swab	-	(−)	
14	Throat Swab	27.25	Flu A (+)	
15	Throat Swab	35.71	Flu A (+)	
16	Throat Swab	26.67	Flu A (+)	
17	Throat Swab	-	(−)	SARS-CoV-2 (+)
18	Throat Swab	28.47	Flu A (+)	
19	Throat Swab	37.78	Flu A (+)	
20	Throat Swab	-	(−)	
21	Throat Swab	30.77	Flu A (+)	
22	Throat Swab	-	(−)	
23	Throat Swab	-	(−)	Adenovirus (+)
24	Throat Swab	-	(−)	Adenovirus (+)

## Data Availability

The original contributions presented in the study are included in the article and Supplementary Materials. Further inquiries can be directed to the corresponding authors.

## Supplementary Materials

The following supporting information can be downloaded at: https://www.mdpi.com/article/10.3390/molecules30234608/s1, Figure S1:Effect of reporter molecule adsorption by magnetic beads after 1 min; Table S1: Nucleic Acid Sequences Used in This Study.

## References

[1] Uyeki T.M., Hui D.S., Zambon M., Wentworth D.E., Monto A.S. (2022). Influenza. Lancet.

[2] Dunning J., Thwaites R.S., Openshaw P.J.M. (2020). Seasonal and pandemic influenza: 100 years of progress, still much to learn. Mucosal Immunol..

[3] Laybourn H.A., Hellemann Polhaus C., Kristensen C., Lyngfeldt Henriksen B., Zhang Y., Brogaard L., Larsen C.A., Trebbien R., Larsen L.E., Kalogeropoulos K. (2024). Multi-omics analysis reveals the impact of influenza a virus host adaptation on immune signatures in pig tracheal tissue. Front. Immunol..

[4] Nagy A., Horvath A., Farkas A., Furi P., Erdelyi T., Madas B.G., Czitrovszky A., Merkely B., Szabo A., Ungvari Z. (2022). Modeling of nursing care-associated airborne transmission of SARS-CoV-2 in a real-world hospital setting. GeroScience.

[5] Laconi A., Fortin A., Bedendo G., Shibata A., Sakoda Y., Awuni J.A., Go-Maro E., Arafa A., Maken Ali A.S., Terregino C. (2020). Detection of avian influenza virus: A comparative study of the in silico and in vitro performances of current RT-qPCR assays. Sci. Rep..

[6] Cassedy A., Parle-McDermott A., O’Kennedy R. (2021). Virus Detection: A Review of the Current and Emerging Molecular and Immunological Methods. Front. Mol. Biosci..

[7] Niu Q., Jiang Z., Wang L., Ji X., Baele G., Qin Y., Lin L., Lai A., Chen Y., Veit M. (2025). Prevention and control of avian influenza virus: Recent advances in diagnostic technologies and surveillance strategies. Nat. Commun..

[8] Gao R., Liu X., Xiong Z., Wang G., Ai L. (2024). Research progress on detection of foodborne pathogens: The more rapid and accurate answer to food safety. Food Res. Int..

[9] Atmar R.L. (2014). Immunological Detection and Characterization. Viral Infections of Humans.

[10] Song H.O., Kim J.H., Ryu H.S., Lee D.H., Kim S.J., Kim D.J., Suh I.B., Choi D.Y., In K.H., Kim S.W. (2012). Polymeric LabChip real-time PCR as a point-of-care-potential diagnostic tool for rapid detection of influenza A/H1N1 virus in human clinical specimens. PLoS ONE.

[11] Mackay I.M., Arden K.E., Nitsche A. (2002). Real-time PCR in virology. Nucleic Acids Res..

[12] Ngoc L.T.N., Lee Y.C. (2024). Current Trends in RNA Virus Detection via Nucleic Acid Isothermal Amplification-Based Platforms. Biosensors.

[13] Hindson B.J., Ness K.D., Masquelier D.A., Belgrader P., Heredia N.J., Makarewicz A.J., Bright I.J., Lucero M.Y., Hiddessen A.L., Legler T.C. (2011). High-Throughput Droplet Digital PCR System for Absolute Quantitation of DNA Copy Number. Anal. Chem..

[14] Huggett J.F. (2020). The Digital MIQE Guidelines Update: Minimum Information for Publication of Quantitative Digital PCR Experiments for 2020. Clin. Chem..

[15] Srivastava P., Prasad D. (2023). Isothermal nucleic acid amplification and its uses in modern diagnostic technologies. 3 Biotech.

[16] Liu M., Hou Y., Cheng Y., Li Z., Zeng J., Li L., Luo J., Shen B.J.I.M. (2025). Recent advances in isothermal amplification techniques coupled with clustered regularly interspaced short palindromic repeat/Cas systems. Interdiscip. Med..

[17] Feng Y., Zhao X., Ye Q., Zou J., Wan Q., Jiang F., Cai Z., Zhang J., Qu X., Huang J. (2025). Isothermal nucleic acid amplification-based biosensors: The next generation analytical toolkit for point-of-care assay of foodborne pathogens. Trends Food Sci. Technol..

[18] Ahmed M.Z., Badani P., Reddy R., Mishra G. (2021). Clustered Regularly Interspaced Short Palindromic Repeats (CRISPR)/Cas Advancement in Molecular Diagnostics and Signal Readout Approaches. J. Mol. Diagn..

[19] Xu D., Wu Q., Yang F., Zhang Q., Jiang Q., Zeng X., Zhang Y., Lv T., Wang J., Li F. (2025). Fast-Flu: RT-RPA-CRISPR/Cas12a assisted one-step platform for rapid influenza B virus detection. Microbiol. Spectr..

[20] Broughton J.P., Deng X., Yu G., Fasching C.L., Servellita V., Singh J., Miao X., Streithorst J.A., Granados A., Sotomayor-Gonzalez A. (2020). CRISPR–Cas12-based detection of SARS-CoV-2. Nat. Biotechnol..

[21] Liu L., Li X., Ma J., Li Z., You L., Wang J., Wang M., Zhang X., Wang Y. (2017). The Molecular Architecture for RNA-Guided RNA Cleavage by Cas13a. Cell.

[22] Tambe A., East-Seletsky A., Knott G.J., Doudna J.A., O’Connell M.R. (2018). RNA Binding and HEPN-Nuclease Activation Are Decoupled in CRISPR-Cas13a. Cell Rep..

[23] Ahmad W., Gong Y., Abbas G., Khan K., Khan M., Ali G., Shuja A., Tareen A.K., Khan Q., Li D. (2021). Evolution of low-dimensional material-based field-effect transistors. Nanoscale.

[24] Sengupta J., Hussain C.M. (2024). Graphene transistor-based biosensors for rapid detection of SARS-CoV-2. Bioelectrochemistry.

[25] Li H., Yang J., Wu G., Weng Z., Song Y., Zhang Y., Vanegas J.A., Avery L., Gao Z., Sun H. (2022). Amplification-Free Detection of SARS-CoV-2 and Respiratory Syncytial Virus Using CRISPR Cas13a and Graphene Field-Effect Transistors. Angew. Chem..

[26] Sun Y., Yang C., Jiang X., Zhang P., Chen S., Su F., Wang H., Liu W., He X., Chen L. (2023). High-intensity vector signals for detecting SARS-CoV-2 RNA using CRISPR/Cas13a couple with stabilized graphene field-effect transistor. Biosens. Bioelectron..

[27] Zhang Z., Hu J.-J., Lin S., Wu J., Xia F., Lou X. (2024). Field effect transistor biosensors for healthcare monitoring. Interdiscip. Med..

[28] Forsyth R., Devadoss A., Guy O.J. (2017). Graphene Field Effect Transistors for Biomedical Applications: Current Status and Future Prospects. Diagnostics.

[29] Chen Z., Wu C., Yuan Y., Xie Z., Li T., Huang H., Li S., Deng J., Lin H., Shi Z. (2023). CRISPR-Cas13a-powered electrochemical biosensor for the detection of the L452R mutation in clinical samples of SARS-CoV-2 variants. J. Nanobiotechnol..

[30] Li J., Tang L., Li T., Li K., Zhang Y., Ni W., Xiao M.-M., Zhao Y., Zhang Z.-Y., Zhang G.-J. (2022). Tandem Cas13a/crRNA-Mediated CRISPR-FET Biosensor: A One-for-All Check Station for Virus without Amplification. ACS Sens..

[31] Hu F., Liu Y., Zhao S., Zhang Z., Li X., Peng N., Jiang Z. (2022). A one-pot CRISPR/Cas13a-based contamination-free biosensor for low-cost and rapid nucleic acid diagnostics. Biosens. Bioelectron..

[32] Xu X., Yu Y., Hu Q., Chen S., Nyholm L., Zhang Z. (2021). Redox Buffering Effects in Potentiometric Detection of DNA Using Thiol-Modified Gold Electrodes. ACS Sens..

[33] Han Y., Li F., Yang L., Guo X., Dong X., Niu M., Jiang Y., Li L., Li H., Sun Y. (2023). Imunocapture Magnetic Beads Enhanced and Ultrasensitive CRISPR-Cas13a-Assisted Electrochemical Biosensor for Rapid Detection of SARS-CoV-2. Biosensors.

[34] Sadighbayan D., Hasanzadeh M., Ghafar-Zadeh E. (2020). Biosensing based on field-effect transistors (FET): Recent progress and challenges. TrAC Trends Anal. Chem..

[35] Sakata T. (2024). Signal transduction interfaces for field-effect transistor-based biosensors. Commun. Chem..

[36] Sun M., Yu Z., Wang S., Qiu J., Huang Y., Chen X., Zhang Y., Wang C., Zhang X., Liang Y. (2025). Universal Amplification-Free RNA Detection by Integrating CRISPR-Cas10 with Aptameric Graphene Field-Effect Transistor. Nano Micro Lett..

[37] Wright A.J., Nasralla H.H., Deshmukh R., Jamalzadeh M., Hannigan M., Patera A., Li Y., Manzo-Perez M., Parashar N., Huang Z. (2024). Nanoscale-localized multiplexed biological activation of field effect transistors for biosensing applications. Nanoscale.

[38] Zheng Y., Zhang C., Zhang Y., Zhou K., Tan P., Liu Y., Duan G., Li H., Chen C., Guo C. (2025). A Nanomaterial-Independent Biosensor Based on Gallium Arsenide High-Electron-Mobility Transistors for Rapid and Ultra-Sensitive Pathogen Detection. ACS Sens..

[39] Feng X., Li P., Xiao M., Li T., Chen B., Wang X., Wang L. (2024). Recent advances in the detection of pathogenic microorganisms and toxins based on field-effect transistor biosensors. Crit. Rev. Food Sci. Nutr..

[40] Meng Q., Li H., Zhao W., Song M., Zhang W., Li X., Chen J., Wang L. (2024). Overcoming Debye screening effect in field-effect transistors for enhanced biomarker detection sensitivity. Nanoscale.

[41] Ding Y., Li C., Tian M., Wang J., Wang Z., Lin X., Liu G., Cui W., Qi X., Li S. (2023). Overcoming Debye length limitations: Three-dimensional wrinkled graphene field-effect transistor for ultra-sensitive adenosine triphosphate detection. Front. Phys..

