# Supplementary material for: A CRISPR Powered Immobilization-Free, Amplification-Free Carbon-Nanotube Field-Effect Transistor (FET) Sensor for Influenza A Virus (IAV)
Source: Molecules. 2025 Nov 30;30(23):4608. doi: 10.3390/molecules30234608 (PMC12692876; doi:10.3390/molecules30234608)
Supplement: Supplementary file 1 [file molecules-30-04608-s001.zip › molecules-3928783-supplementary.pdf]

1 Screening of Conserved Sequences of Influenza A Virus (IAV)

Genomic sequence data of the IAV M gene was downloaded from the NCBI Influenza Virus Database. The downloaded sequences were aligned using the MAFFT program, and the alignment results were visualized with Jalview software to screen for appropriate highly conserved sequences. Detailed information of the conserved sequences is provided in Table 1. Based on the conserved sequences of IAV and the designed RT-RAA primers, crRNA was designed between the forward and reverse primers. The crRNA sequence consists of two parts: a scaffold sequence and a target sequence. First, a 28 bp fragment was selected as the target sequence of crRNA. Subsequently, the crRNA promoter sequence (5'-GGGAUUUAGACUACCCCAAAAACGAAGGGGACUAAAAC-3') was added to the 5' end of the target sequence. Annealing primers were designed using the single-stranded DNA sequence of crRNA as the amplification template for in vitro transcription and synthesis of crRNA. The upstream primer (crRNA-F) was designed to contain the T7 promoter sequence and 5'-GATTTAGACTACCCCAA-3'. The downstream primer (crRNA-R) was designed to include the reverse complement of the 20 bp target sequence.。

Table S1 Nucleic Acid Sequences Used in This Study

Name	sequence (5'→3')
Conserved sequences of IAV	ATGAGTCTTCTAACCGAGGTCGAAACGTACGTTCTCTC TATCATTCCATCAGGCCCCCTCAAAGCCGAGATCGCGC AGAGACTTGAAGATGTCTTTGCAGGGAAGAACACAG ATCTTGAGGCTCTCATGGAATGGCTAAAGACAAGACC AATCCTGTCACCTCTGACTAAGGGGATTTTAGGGTTTG TGTTCACGCTCACCGTGCCCAGTGAGCGAGGACTGCA GCGTAGACGCTTTGTCCAAAATGCTTGGGAATGG
PCR-F	CCGAGGTCGAAACGTACGTT
PCR-R	GTCTACGCTGCAGTCCTCG
IAV-qPCR-F	CCGAGGTCGAAACGTACGTT
IAV-qPCR-R	GTCTACGCTGCAGTCCTCG
IAV-qPCR-Probe	ACGCTCACCGTGCCCAGTGA
T7-crRNA-F	TAATACGACTCACTATAGGGGATTTAGACTACCCCAA
crRNA-1	GGGATTTAGACTACCCCAAAAACGAAGGGGACTAAAA CTCTTTAGCCATTCCATGAGAGCCTCAAG
crRNA1-R	CTTGAGGCTCTCATGGAATG
crRNA-2	GGGATTTAGACTACCCCAAAAACGAAGGGGACTAAAA CTTGTCTTTAGCCATTCCATGAGAGCCTC
crRNA2-R	GAGGCTCTCATGGAATGGCT
crRNA-3	GGGATTTAGACTACCCCAAAAACGAAGGGGACTAAAA CTCTTGTCTTTAGCCATTCCATGAGAGCC
crRNA3-R	GGCTCTCATGGAATGGCTAA
crRNA-4	GGGATTTAGACTACCCCAAAAACGAAGGGGACTAAAA CGGTCTTGTCTTTAGCCATTCCATGAGAG
crRNA4-R	CTCTCATGGAATGGCTAAAG
crRNA-5	GGGATTTAGACTACCCCAAAAACGAAGGGGACTAAAA CATTGGTCTTGTCTTTAGCCATTCCATGA
crRNA5-R	TCATGGAATGGCTAAAGACA

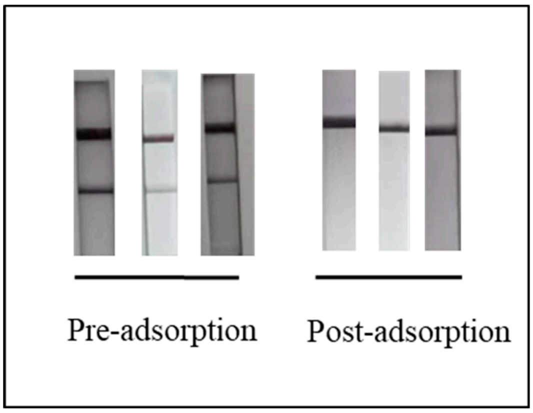

Figure S1. Effect of reporter molecule adsorption by magnetic beads after 1 min

Before adsorption, the reporter molecules produced two bands when passing through the test strip. After 1 minute of magnetic bead adsorption, only one band remained, indicating a significant adsorption effect of the magnetic beads
